# Reducing instruments in a vitrectomy surgical tray: cost savings and results from a major academic hospital

**DOI:** 10.1186/s40942-020-00215-2

**Published:** 2020-06-18

**Authors:** Jacob D. Grodsky, Christos N. Theophanous, Sidney A. Schechet, Peter B. Veldman, Seenu M. Hariprasad

**Affiliations:** 1 Department of Ophthalmology, Saint Louis University Eye Institute, 1755 South Grand Blvd., Saint Louis, MO 63104 USA; 2 Department of Ophthalmology and Visual Science, University of Chicago Medicine, 5741 S. Maryland Avenue, S-439, Chicago, IL 60637 USA

## Abstract

**Background:**

Unused or rarely used instruments in standard surgical trays can unnecessarily increase costs. Prior studies have demonstrated the practicality and cost savings of reduced instrument tray sizes in various subspecialties. This study describes results and estimated cost savings from a reduced instrument tray used for vitrectomy surgery at a large, tertiary academic medical center.

**Methods:**

Common usage patterns of vitrectomy instruments by one retina surgeon were reviewed and a reduced instrument vitrectomy tray was created and implemented in successive vitrectomy surgeries. Need for opening the previously utilized larger tray was recorded. Estimated cost savings of the new trays were calculated based upon per instrument sterilization, processing, and instrument replacement costs.

**Results:**

New vitrectomy trays including just 7 instruments (89% reduction compared to original trays) were created and implemented in 189 successive cases. The original tray was never opened. Estimated cost savings from saved sterilization and processing resources is approximately $9588 per year. Assuming 5- and 10-year lifespan per instrument, annual cost avoidance is projected at $7886 and $15,772, respectively. Other indirect benefits relevant to healthcare quality were also noted.

**Conclusion:**

A reduced instrument tray can be successfully implemented for vitrectomy surgery and can result in significant indirect benefits as well as direct cost savings from reduced sterilization costs. Our study highlights the substantial impact made by evaluating the usage pattern and making appropriate instrument tray changes for just one retina surgeon. Applying these same methods to other surgeons and specialties can have significant implications on healthcare costs and quality.

## Background

Surgical trays that contain unused instruments can lead to unnecessary costs for hospitals [1–3]. Specifically, unused instruments undergo sterilization between each case, resulting in both direct labor costs and more rapid wear and tear of instruments [1, 2, 4, 5]. In addition, unpacking large trays can add to case turnover time and increase time spent counting items both before and after each case [2, 4, 5]. As a result, reducing instrument tray size by eliminating unused or rarely used instruments may represent a cost savings opportunity.

Previous studies have demonstrated that standard surgical trays often contain numerous rarely used instruments [1, 3]. Studies also demonstrate that a reduction in main instrument tray size led to a reduction in instrument assembly time, setup time, and increased surgeon and scrub personnel satisfaction [3]. Such reductions in surgical tray instrumentation can generate cost savings, which have been estimated in several surgical specialties. For instance, a 70% instrument reduction in surgical trays used for both minimally invasive spine surgery and deep brain stimulation surgery demonstrated a cost savings of approximately $60,000 per year [2]. A study from Canada demonstrated that reducing redundant instruments from trays for five common otolaryngology procedures saved an estimated CAD 13,000 annually [5]. Additional cost savings can be seen by avoiding instrument purchase and replacement costs through reduced surgical trays and delaying wear and tear of instruments [6].

To our knowledge, no study has used similar methodology to analyze any ophthalmic surgical trays, create and implement a reduced instrument tray, and calculate expected cost savings. A thorough review of the current literature has revealed studies from various other medical fields that analyzed and reduced the number of instruments in surgical trays (Table 1). In this study, we identified commonly unused instruments included in our standard vitrectomy tray and sought to create a reduced instrument tray. After creating and implementing this new tray into our surgical workflow, we then recorded the need for opening additional instruments due to inability to complete a case with the newly created reduced instrument tray. Lastly, we calculated estimated annual cost savings based on these changes.

**Table 1:** Reported reductions in surgical tray instrumentation from selected studies

Study	Surgical Field	Percent Instrument Reduction (%)
Byrnes 2017 [7]	Gynecology	28.4
Chin 2014 [1]	Otolaryngology	57
Farrelly 2017 [6]	Pediatric surgery	39.5 (*General)*
		10.1 *(Urology)*
		17.1 *(Orthopedics)*
		92.7 *(Spine)*
		32.3 *(Neurosurgery)*
Farrokhi 2015 [2]	Minimally invasive spinal surgery	70
John-Baptiste 2016 [5]	Otolaryngology	56.4
Nast 2019 [8]	Pediatric urology	38
Wannemuehler 2015 [3]	Otolaryngology	54

## Methods

**Fig. 1:**
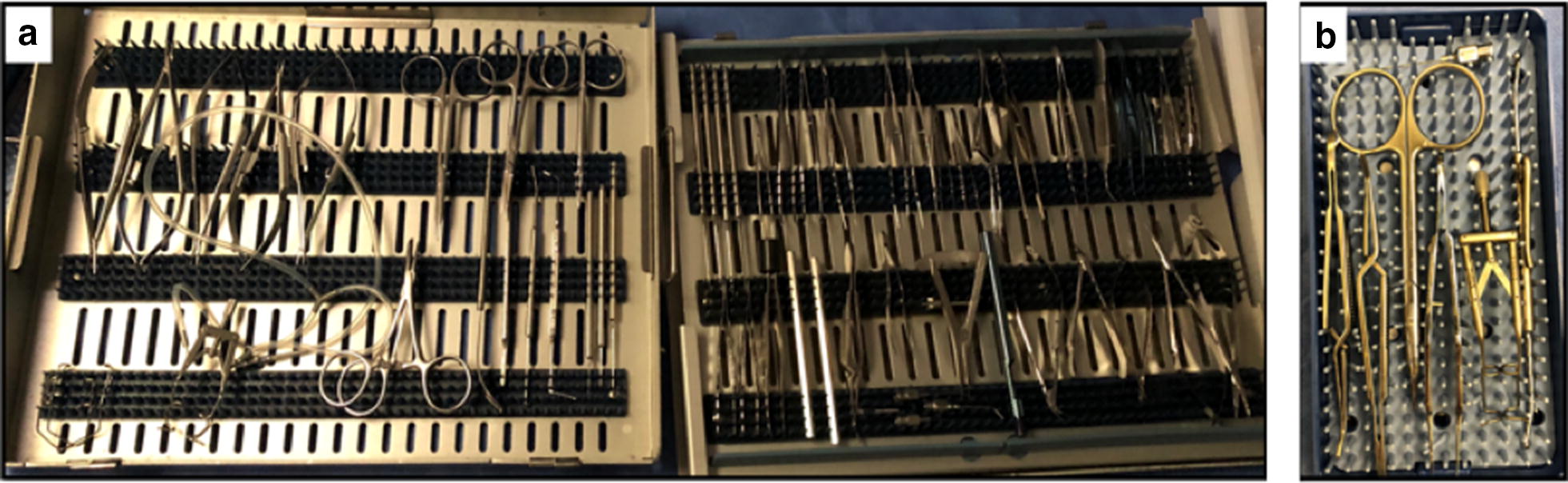
**a** Standard vitrectomy tray containing 64 instruments; **b** Reduced instrument vitrectomy tray containing 7 instruments

**Fig. 2:**
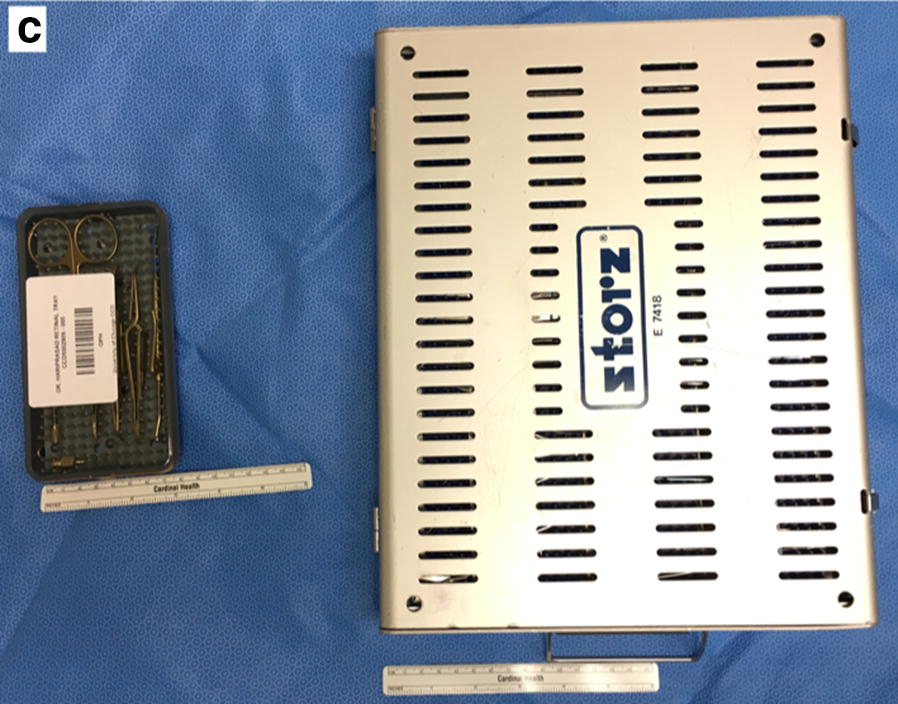
**c** Size comparison of reduced (left) and standard (right) vitrectomy trays

This study designed and implemented a new reduced instrument vitrectomy tray utilized by one retina surgeon. Instruments contained in the standard vitrectomy tray were reviewed for redundancy, and those utilized in the majority of vitrectomy cases were included in the new reduced instrument tray. Images of the standard and reduced trays are shown in Figs. 1, 2.

Unnecessary instruments not frequently used in vitrectomy surgery that had been added to the standard tray over previous years were excluded from the new reduced tray (Table 2). IRB approval is not required at our institution for studies of this nature. In addition, it was not appropriate or possible to involve patients or the public in this work.

**Table 2:** Instruments included in standard and reduced instrument vitrectomy trays

Standard large vitrectomy tray (n = 64)		Newly created reduced vitrectomy tray (n = 7)	
Instrument	Quantity	Instrument	Quantity
Westcott tenotomy scissor, curved blunt	2	Lieberman, open wire, angled, 45-degree speculum	1
Vannas scissors, curved	1	Scleral depressor	1
Corneal scissors, left	1	Castroviejo suturing forceps 0.3	1
Corneal scissors, right	1	Straight stevens scissors	1
Plug holder	4	25 G aspirating canula	1
19G cannula BSS	2	Plug holder	2
Castroviejo caliper, straight	1		
Scleral depressor	1		
Beaver knife handle	2		
Bipolar cautery forceps	1		
Bishop harmon forceps with teeth	2		
Bishop harmon forceps without teeth	2		
Titanium tyer, short	1		
Titanium tyer, long	1		
Tyer, harms	2		
Bonaccolto forceps	2		
Colibri forceps	1		
Cross action forceps	1		
Bonn suturing forceps	2		
Castroviejo fine suturing forceps 0.12	2		
Castroviejo suturing forceps 0.3	2		
Nugent forceps	2		
Retaining nut for blackflush	1		
Gass retinal hook with hole	4		
Muscle hook	1		
Sinskey hook	1		
Kuglen hook	1		
Cyclodialysis spatula	1		
Scleral marker	1		
Knife handle #3, 5″	1		
Short schepens orbital retractor	1		
Hemostat	2		
Curved stevens tenotomy scissors	1		
Straight stevens scissors	1		
Straight, sharp stevens scissors	1		
Iris scissors	1		
Wire speculum	2		
Straight, locking, micro Barraquer needleholder	2		
Non-locking, micro Barraquer needleholder	1		
Locking needleholder	2		
Lieberman, open wire, angled, 45-degree speculum	1		
25 G blunt canula	1		
Lieberman aspirating speculum with silicone tubing	1		

We estimated the expected cost savings of implementing the smaller trays based on internal institution costs for instrument sterilization. Utilizing the general formula published by Farrokhi et al., we estimated internal sterilization costs per instrument by dividing the total costs for 1 year of instrument sterilization for the University of Chicago Medicine Central Sterile Processing (CSP) department between July 1, 2017 and June 30, 2018 by the total number of surgical instruments sterilized in that same time period [2]. The total costs reflected the entire budget of the department, including both physical resource costs, labor, instrument repair and replacement, and necessary utilities and supplies for instrument cleaning. Estimated cost savings per instrument in our reduced vitrectomy tray were annualized by multiplying the number of instruments excluded from the reduced vitrectomy trays by the total number of vitrectomy cases performed using only the new tray during a 12-month time period.

Data related to instrument cost avoidance was obtained via quotes from University of Chicago vendors. Individualized instrument costs were obtained and used to calculate annual instrument cost avoidance. Recognizing that instruments have varying life expectancies, annual instrument cost avoidance savings are based on a range of replacing instruments every 1, 5, or 10 years, consistent with previously published analyses [6].

The reduced instrument trays were implemented and opened for all vitrectomy cases performed by one retina surgeon between July 15, 2018 and July 14, 2019. During this 12-month period, the original large vitrectomy tray was kept in circulation to be opened only if additional instrumentation was needed during cases. The number of cases requiring instruments from the larger tray during this period was recorded.

## Results

The original standard vitrectomy trays included 64 instruments. Of these, 7 instruments were used in a majority of cases and were included in the reduced trays, resulting in an 89% reduction of instruments. Reduced trays measured 8.4 cm × 15.8 cm and weighed 0.22 kg compared to standard trays that measured 25.8 cm × 33.0 cm and weighed 3.0 kg (84% reduction by surface area on scrub table and 93% reduction by weight).

During our study duration, we had 189 successive vitrectomy cases. Indications for vitrectomy and surgical procedures performed are shown in Table 3.

**Table 3:** Indications and surgical procedures performed

Primary indication for pars plana vitrectomy	Quantity	Intraoperative procedures performed	Quantity
Tractional retinal detachment	90	23 g PPV	183
Rhegmatogenous retinal detachment	25	25 g PPV	5
Epiretinal membrane	32	20 g PPV	1
Vitreous hemorrhage (± retinal hole or tear)	14	Membrane peel	162
Macular hole	14	Endolaser	112
Dislocated/retained lens fragments	7	Air/gas fluid exchange	104
Silicone oil removal	4	C_3_F_8_ instillation	32
Uveitis with vitreous debris	1	Silicone oil instillation/removal	22
Endophthalmitis	2	Subtenon’s steroid injection	15
		Retinectomy	3
		Lensectomy	7

During these 189 cases, the reduced instrument tray was used for the entire case in all 189 cases, and the large original instrument tray was opened in zero cases.

In the 12 months preceding the study, the CSP department sterilized a total of 5,254,400 instruments. During that time, total costs for the CSP department were $5,886,465.50. Instrument sterilization cost was calculated to be $0.89 per instrument [2]. As the reduced instrumentation tray excluded 57 previously included instruments, each vitrectomy case utilizing only the reduced instrument tray resulted in an estimated cost savings of $50.73. With these estimates, the total annual cost savings of the new vitrectomy sets in 189 cases is approximately $9588.

Using data obtained from our institution’s preferred instrument supplier, the 57 instruments eliminated from the reduced instrument tray cost $9857 to replace. Our institution currently has 8 of our original vitrectomy trays as well as 8 of our new mini vitrectomy trays in circulation. By eliminating 57 instruments from each of our original trays, this results in a projected annual cost avoidance of $7886 and $15,772 in replaced vitrectomy instruments, assuming a 10-year or 5-year instrument lifespan, respectively.

## Discussion

This study demonstrates that a reduced instrument tray can be successfully implemented for vitrectomy surgeries and result in significant estimated cost savings. We achieved an 89% reduction in instruments included in the standard vitrectomy kit when creating our new kit. Opening the larger original tray was never needed during 189 successive vitrectomy cases for a variety of indications.

Cost savings per instrument calculated in our study are roughly in line with previously published sterilization cost estimates by Farrokhi et al. [2] They are, however, higher than a study by Stockert et al. previously performed at our institution in which sterilization cost per instrument ranged from $0.12 to $0.51 [4]. These values were calculated through estimations and analysis of CSP labor costs, instrument depreciation, CSP department utilities, and repair costs. Elements of their methodology have been cited by several other studies [5]. Since the study was performed, the CSP department sterilizes approximately 57% more instruments per year and granular data on the elements included in the study could not be obtained. Our cost estimates also include some overhead costs for the department that may not have been captured by the Stockert study, which explains the higher per instrument cost.

Aside from cost savings, further indirect benefits of reducing the size of vitrectomy trays were noted. Our surgical staff noticed a decrease in turnover time between cases, less time needed by the surgical scrub nurse to lay out and organize the instruments, and faster response time for the scrub nurse to hand over instruments requested by the surgeon during the case. Overall, there was a significant increase in subjective satisfaction by both the operating room staff and the retina surgeon after the implementation of the smaller instrument tray. Furthermore, we believe that the fewer number of instruments needed for each case decreases wear and tear on the unused instruments. Our study highlights the substantial impact made by evaluating the usage pattern and making appropriate surgical instrument tray changes for just one surgeon. Applying this same evaluation and adjustment methods to other surgeons and specialties can have significant implications on healthcare costs and quality.

A limitation of this study is that it focuses solely on one surgeon’s experience in reducing the number of instruments included in one specific surgical tray. Different surgeons and institutions may vary widely in their instrument utilization patterns. Specific amounts and types of instruments included in the surgical tray before removing those that are unnecessary may also vary widely amongst institutions. Cost savings at other institutions will likely also differ from our results based on their individual sterilization processes, instrument wear and tear, and replacement processes. We were also unable to quantify the cost saving impact of factors such as the labor cost required to count, decontaminate, and pack the surgical trays as well as the depreciation of instruments, CSP washers, and sterilization machines.

## Conclusions

We have demonstrated that there are significant direct and indirect benefits of reducing the number of instruments included in surgical trays. Our study highlights that these benefits extend not only to the surgeon, but also to the surgical staff and the hospital. Our study may also suggest opportunities for cost savings in other ophthalmologic subspecialties and for other types of ocular surgery through a systematic evaluation of instrument use patterns and streamlined standardized surgical trays. Based on our experience, we recommend that hospitals partner with their surgeons to assess and reduce surgical tray size in order to cut operating costs and reduce unnecessary time in the operating room.

## Data Availability

The datasets used and/or analyzed during the current study are available from the corresponding author on reasonable request.

## Abbreviation

CSP: Central Sterile Processing
